# Majorana fermions in the nonuniform Ising-Kitaev chain: exact solution

**DOI:** 10.1038/s41598-017-01413-z

**Published:** 2017-05-03

**Authors:** Boris Narozhny

**Affiliations:** 1 Institut für Theorie der Kondensierten Materie, Karlsruher Institut für Technologie, 76128 Karlsruhe, Germany; 2 National Research Nuclear University MEPhI (Moscow Engineering Physics Institute), Kashirskoe shosse 31, 115409 Moscow, Russia

## Abstract

A quantum computer based on Majorana qubits would contain a large number of zero-energy Majorana states. This system can be modelled as a connected network of the Ising-Kitaev chains alternating the “trivial” and “topological” regions, with the zero-energy Majorana fermions localized at their interfaces. The low-energy sector of the theory describing such a network can be formulated in terms of leading-order couplings between the Majorana zero modes. I consider a minimal model exhibiting effective couplings between four Majorana zero modes – the nonuniform Ising-Kitaev chain, containing two “topological” regions separated by a “trivial” region. Solving the model exactly, I show that for generic values of the model parameters the four zero modes are localized at the four interface points of the chain. In the special case where additional inversion symmetry is present, the Majorana zero modes are “delocalized” between two interface points. In both cases, the low-energy sector of the theory can be formulated in terms of the localized Majorana fermions, but the couplings between some of them are independent of their respective separations: the exact solution does not support the “nearest-neighbor” form of the effective low-energy Hamiltonian.

## Introduction

Physicists have been fascinated with Majorana fermions ever since their discovery^1, 2^ in 1937, when Ettore Majorana found a completely real (i.e. not containing complex coefficients) representation of the Dirac equation. The solutions of the Majorana equation describe neutral fermions – particles that obey the Fermi statistics, but at the same time are their own antiparticles. Whether they exist in nature as elementary particles is still an open question. It has been hypothesized that neutrinos might be Majorana fermions. This hypothesis could be experimentally confirmed by observation of an elusive process known as the neutrinoless double beta decay^3^, which is the focus of considerable experimental efforts.

Recently, it has become possible to imitate the ideas of the relativistic field theory in solids. Following the success of graphene research, further novel materials have been identified as Dirac^4^ and Weyl^5, 6^ semimetals. These materials exhibit a finite number of band crossings at the Fermi level (the so-called Dirac and Weyl points)^7, 8^. To a good approximation, low-energy excitations near these points are characterized by the (quasi)-relativistic spectrum allowing one to observe phenomena previously belonging to the realm of high energy physics, such as the Bell-Adler-Jackiw chiral anomaly^9–12^.

At the same time, signatures of Majorana fermions were found in nanowires with proximity-induced superconductivity^13–20^. While the physics of such systems is rather complex, the effective low-energy Hamiltonian describing the nanowire is essentially that of the one-dimensional (1D) *p*-wave superconductor, i.e. the continuous limit of the Kitaev model^21–24^. The 1D lattice model proposed by Kitaev^21^ exhibits a quantum phase transition between two gapful (massive) phases, known as the “trivial” and “topological”. The “trivial” phase is characterized by a single non-degenerate ground state, while the “topological” phase possesses a ground state that is nearly doubly degenerate: for any finite-size, open chain the difference between the energies of the lowest-lying excited state and the ground state is exponentially small, ∝$\exp (\,-\,L/{\ell }_{0})$, in the length of the chain *L* (here ${\ell }_{0}$ is a certain correlation length defined below). In the thermodynamic limit, the energy difference vanishes and the ground state becomes truly degenerate. This is a manifestation of a well known theorem in statistical physics^25^: spontaneous symmetry breaking and the corresponding vacuum degeneracy may only occur in the thermodynamic limit. The lowest-lying excitation in the “topological” phase of the Kitaev model is a fermion with a wave function that is nonzero (with exponential accuracy) only near the two edges of the chain^21^. This fermion can be described in terms of two Majorana fermions, one at each edge^21, 26^. It is these objects, known as the “Majorana bound states”^13, 20, 24^ or “Majorana zero modes”^14, 17, 19^, that have been observed.

Arguably the main driving force behind the pursuit of Majorana zero modes in solids is the possibility of applications to quantum computing^21, 27, 28^. The basic building block of a quantum computer, the qubit, can be realized as a coupled system of four spatially separated zero-energy Majorana states^26, 29, 30^. It is expected that a Majorana qubit would have a rather long coherence time due to its topological nature^29, 31^. Quantum computer can then be envisioned as a connected network of such qubits. Certain logical operations in such a computer can also be performed topologically by means of braiding (or adiabatic interchange) of Majorana fermions^29, 32^.

Alternatively, one can search for Majorana fermions in manifestly discreet systems^33–36^. For instance, one may engineer the Majorana bound states using Josephson qubits^37, 38^ to build an artificial spin chain^33, 34^ that is designed to be an experimental realization of the 1D quantum Ising model^39–43^. The quantum Ising chain with open boundary conditions is formally equivalent to the Kitaev chain^21, 28, 34, 42, 43^ (note, that the two models do not enjoy the same level of the topological protection^21, 44^). This equivalency is based on the Jordan-Wigner transformation^45^ that is commonly used in 1D theories to express the spin-1/2 operators in terms of creation and annihilation operators of spinless fermions^40^. In fact, the original solution^43^ of the 1D quantum Ising model was based on the consequent application of the Jordan-Wigner transformation and the Bogolyubov transformation^46^, mapping the model onto a system of free fermions^42^. The simplicity of the resulting physical picture may be deceptive, since both the Jordan-Wigner and Bogolyubov transformations are nonlocal^44^. Although the original Hamiltonian contains only nearest-neighbor couplings, the model may develop long-range correlations. In fact, the ground state of the open-ended chain is characterized by the “end-to-end” correlation function^43^ that vanishes in the “trivial” phase (in the thermodynamic limit), but remains finite in the “topological” phase. This result can be interpreted in terms of a nonlocal fermion operator that is a linear combination of the Jordan-Wigner fermions at both ends of the chain. The lowest excited state of the open-ended chain in the “topological” phase (i.e. the state that is nearly degenerate with the ground state) possesses a similar structure. The wave function of this state decays exponentially away from the chain ends and hence can be represented as a linear combination of the two states localized at either end of the chain. The existence of such edge states has been known for a very long time^42^, but they were not interpreted in terms of Majorana fermions and related to the quantum information theory before the work of Kitaev^21^.

A quantum computer based on Majorana qubits would contain a large number of Majorana zero modes. Whether the device will be built using the nanowires^22–24^ or the artificial spin chains^33, 34^, one can envision the effective model of the system as a connected network of the Ising-Kitaev chains alternating the “trivial” and “topological” regions, with the zero-energy Majorana fermions localized at their interfaces^27^. The low-energy sector of such a theory can be formulated in terms of leading-order couplings between the Majorana zero modes^27, 30, 47, 48^. These couplings are often chosen based on physical intuition. Given the nonlocal relation between the Majorana zero modes and the Kitaev (or Jordan-Wigner) fermions, it is desirable to test that intuition against a rigorous solution of a representative microscopic model. This is the principle goal of the present work.

**Figure 1:**
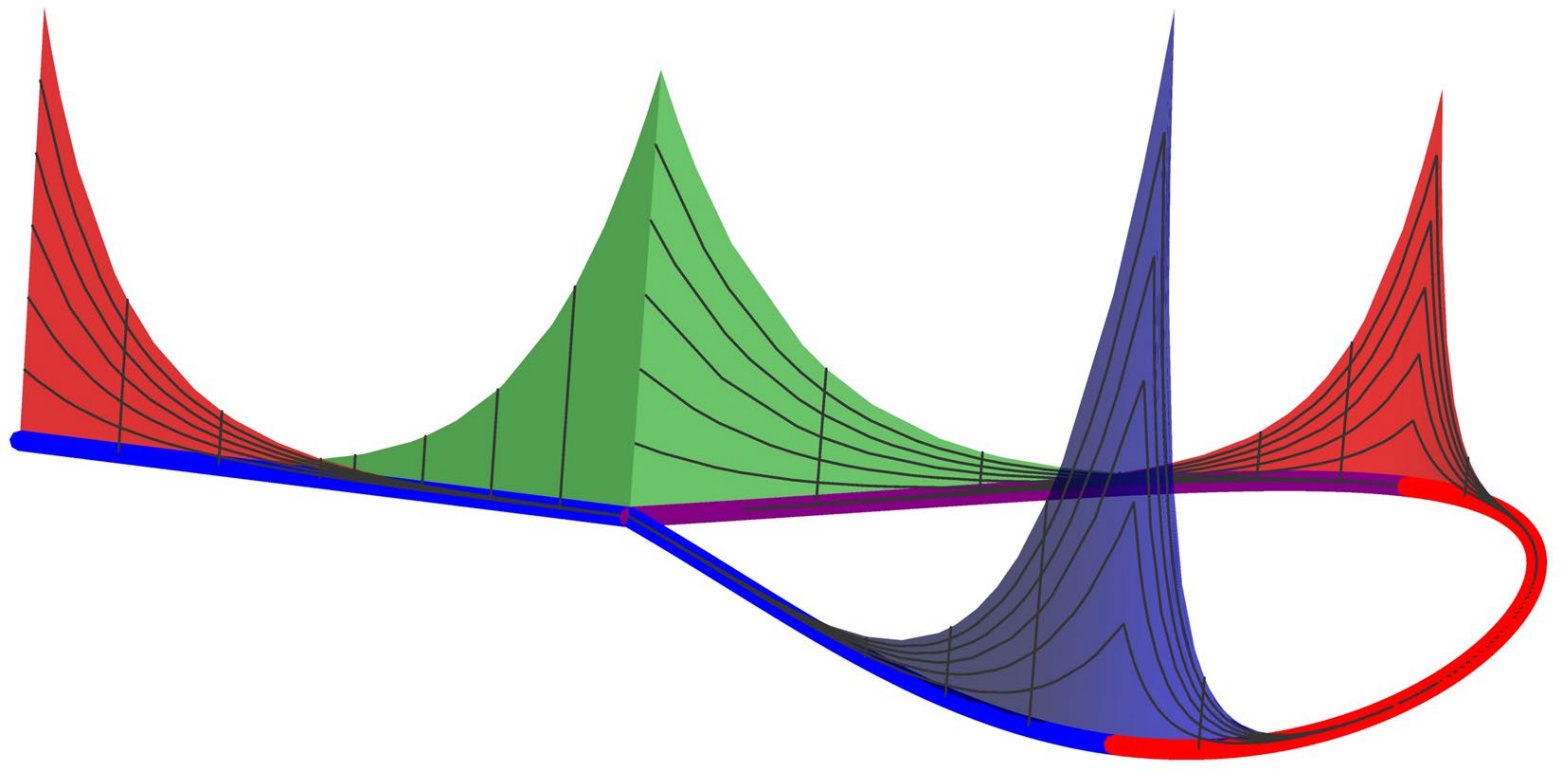
A three-dimensional illustration of the Majorana bound states in the Kitaev model with a T-junction. The chain contains two “topological” regions (the blue line, with *N*_1_ sites, and the purple line, with *N*_2_ sites) and one “trivial” region (the red line, with *M* sites). The T-junction is located at the site *N*_0_. The peaks represent the absolute values of the real-space amplitudes of the Majorana zero modes calculated for *N*_1_ = *M* = 20, *N*_2_ = 10, and *N*_0_ = 11. The model exhibits four Majorana zero modes (corresponding to the nearly four-fold degeneracy of the ground state). Two of them are localized at a single interface point each: the dark blue at the site *N*_1_ and the green at the T-junction. Note, that this amplitude is spread over only two (out of three) branches at the junction. The remaining two zero modes are delocalized between two interface points, the sites 1 and *N*_1_ + *M*. One of them is illustrated by the red peaks.

In this paper I consider a minimal model exhibiting effective couplings between Majorana zero modes – the nonuniform Ising-Kitaev chain, containing two “topological” regions separated by a “trivial” region. Based on the common intuition, one would expect that this model possesses four Majorana zero modes, each localized at one of the four interface points of the chain^27, 47^ (i.e. the two chain ends and two edges of the “trivial” region). I present the exact solution of the model and identify the region of model parameters where the above expectation is indeed fulfilled. However, the exact solution also exhibits situations where the intuitive expectation is *not* fulfilled. In particular, inversion symmetry (in the case where the two “topological” regions are identical) leads to “delocalization” of the Majorana zero modes between two interface points. While one can use a basis rotation to express the low-energy sector of the theory in terms of four localized Majorana operators, the corresponding states will no longer be the eigenstates of the model. The low-energy Hamiltonian will then contain effective couplings between some of these modes that are independent of their respective separations. I also demonstrate that the symmetric case in not the only situation exhibiting the “delocalization” of the Majorana bound states. As an example, I show that the “delocalization” may also occur in the variant of the model, where one of the chain ends is coupled to one of the intermediate sites forming a T-junction (or a Y-junction), see Fig. 1.

## Results

### The nonuniform Ising-Kitaev chain

The open-ended, nonuniform quantum Ising chain is described by the Hamiltonian

1 $$\widehat{H}=-\,J\sum _{n=1}^{L-1}{\hat{\sigma }}_{n}^{x}{\hat{\sigma }}_{n+1}^{x}-\sum _{n=1}^{L}{h}_{n}{\hat{\sigma }}_{n}^{z},$$

where ${\hat{\sigma }}_{n}^{i}$ are the Pauli matrices corresponding to a spin 1/2 residing on the site *i*. Using the Jordan-Wigner transformation^40, 45^, this model can be mapped onto a variant of the Kitaev chain^21, 28, 40, 41^

2 $$\hat{H}=-J\sum _{n=1}^{L-1}({\hat{c}}_{n}^{\dagger }-{\hat{c}}_{n})({\hat{c}}_{n+1}^{\dagger }+{\hat{c}}_{n+1})-2\sum _{n=1}^{L}{h}_{n}({\hat{c}}_{n}^{\dagger }-{\hat{c}}_{n})({\hat{c}}_{n}^{\dagger }+{\hat{c}}_{n}).$$

The model originally considered by Kitaev^21^ maps onto the variant of the quantum Ising model containing also the ${\hat{\sigma }}_{n}^{y}{\hat{\sigma }}_{n+1}^{y}$ couplings (the XY model in a transverse field^39, 42^). However, it is well known^39, 41^ that as long as the exchange constants in the *xx* and *yy* terms are not identical, the two models are in the same universality class. The model (2) exhibits all of the essential features of the original Kitaev chain and constitutes a representative model for studies of the Majorana zero modes^28^.

**Figure 2:**
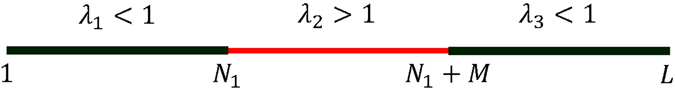
The nonuniform Ising-Kitaev chain split into two “topological” (dark green) and one “trivial” (red) regions. The first “topological” region is characterized by the parameter *λ*_1_ < 1 and occupies the left part of the chain, $1\leqslant n\leqslant {N}_{1}$. The next *M* sites are occupied by the “trivial” phase with *λ*_2_ > 1. The remaining portion of the chain of the length ${N}_{2}=L-{N}_{1}-M$ is occupied by the second “topological” region with *λ*_3_ < 1.

In this paper I focus on the minimal model supporting effective couplings between Majorana zero modes choosing the applied field *h*_*n*_ to be piece-wise uniform (see Fig. 2 for illustration)

3 $${h}_{n}=\{\begin{array}{cc}{h}_{1} < J, & 1\leqslant n\leqslant {N}_{1},\\ {h}_{2} > J, & {N}_{1}+1\leqslant n\leqslant {N}_{1}+M,\\ {h}_{3} < J, & {N}_{1}+M+1\leqslant n\leqslant L.\end{array}$$

In this case, the chain is split into three regions such that the two “topological” regions (of the length *N*_1_ and *N*_2_ = *L* − *N*_1_ − *M*) are separated by the “trivial” region of the length *M*. Since physical properties of the model are determined by the ratios of the applied fields to the exchange coupling *J*, it is convenient to factor out the exchange constant *J* introducing the parameters

4 $${\lambda }_{i}={h}_{i}/J,\,{\lambda }_{1},{\lambda }_{3} < 1,\,{\lambda }_{2} > 1.$$

The finite-size, open-ended lattice model (1)–(2) is exactly solvable (see Methods). The diagonal form of the Hamiltonian (2) is given by

5 $$\widehat{H}=2J\sum _{k=1}^{L}{ {\mathcal E} }_{k}{\hat{\eta }}_{k}^{\dagger }{\hat{\eta }}_{k}-J\sum _{k=1}^{L}{ {\mathcal E} }_{k}-J\sum _{n=1}^{L}{\lambda }_{n}.$$

The first term in Eq. (5) describes the excitation spectrum of the model in terms of free fermion operators ${\hat{\eta }}_{k}$. The two remaining terms yield the ground state energy. For an arbitrary choice of *λ*_*n*_, the energies ${ {\mathcal E} }_{k}$ can be found numerically with arbitrary precision. In the specific case (3), the model can also be solved analytically. Below I present the results of the analytic solution and compare them to the numerical results.

### Nearly degenerate ground states

**Figure 3:**
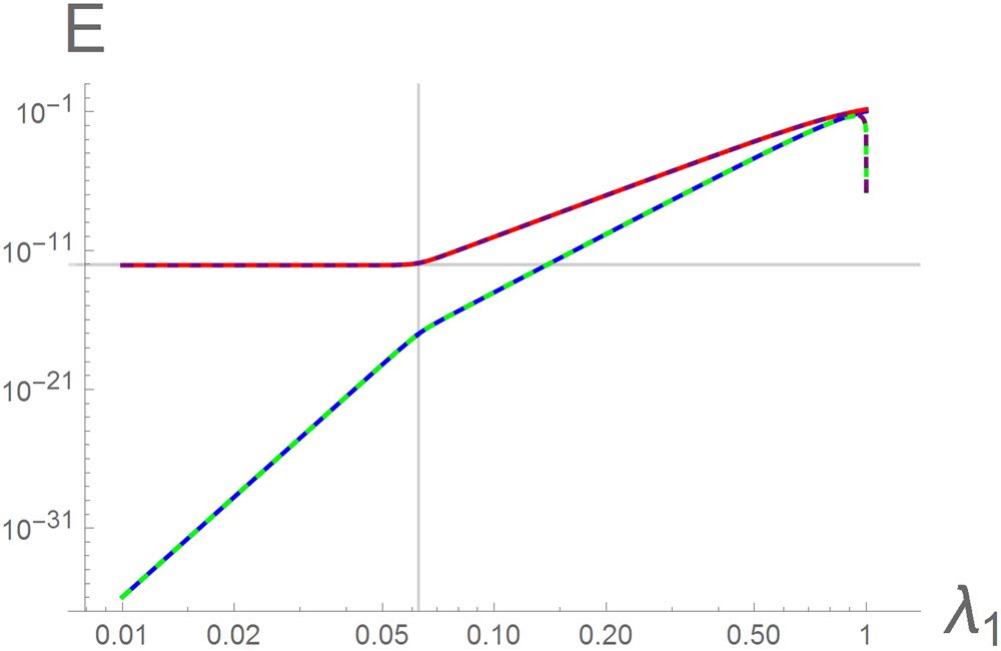
Energy eigenvalues ${ {\mathcal E} }_{\mathrm{1,2}}$ of the two lowest-lying excited states of the Ising-Kitaev chain (1)–(2) in the piece-wise uniform applied field (3) as a function of *λ*_1_ = *λ*_3_ with *λ*_2_ = 4 for *N*_1_ = 10, *M* = 20, and *N*_2_ = 14. The solid curves represent the result of the exact numerical diagonalization. The dashed lines represent the analytic solutions to Eqs (34) and (35). The vertical grid line corresponds to ${\lambda }_{1}^{2{N}_{1}}={\lambda }_{2}^{-2M}$, in this particular case, *λ*_1_ = 1/16. On the right side of this line the green dashed line corresponds to Eq. (6) and the purple – to Eq. (7). On the left side the green dashed line represents Eq. (9), the purple – Eq. (8). The horizontal grid line corresponds to $E={\lambda }_{2}^{-M}={4}^{-20}$.

**Figure 4:**
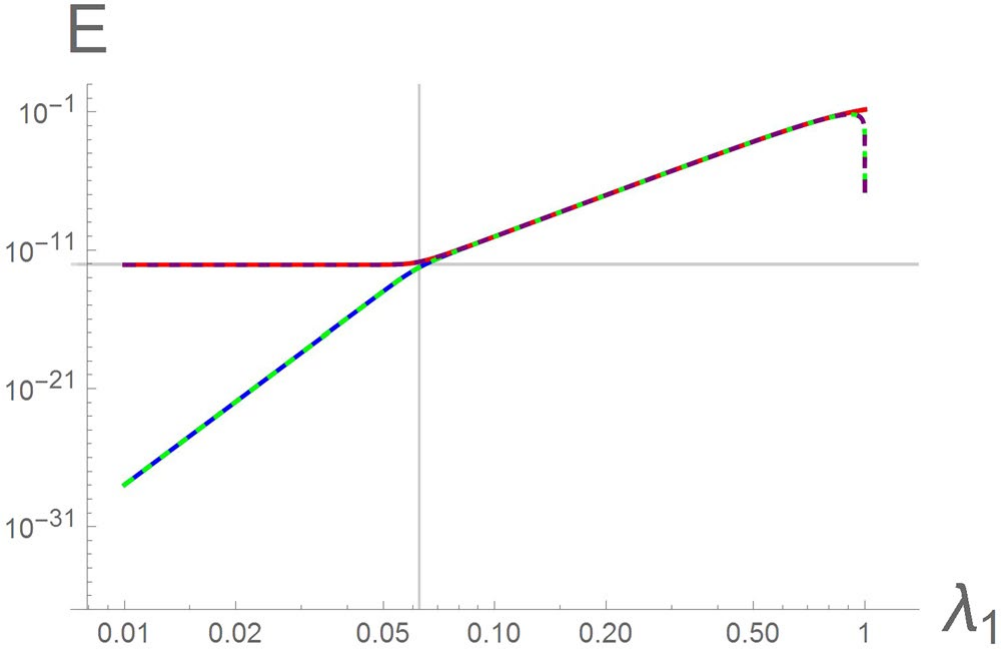
Energy eigenvalues ${ {\mathcal E} }_{\mathrm{1,2}}$ of the two lowest-lying excited states of the symmetric Ising-Kitaev chain (1)–(2) in the piece-wise uniform applied field (3) as a function of *λ*_1_ = *λ*_3_ with *λ*_2_ = 4 for *N*_1_ = *N*_2_ = 10 and *M* = 20. The solid curves represent the result of the exact numerical diagonalization. The dashed lines represent the analytic solutions to Eqs (34) and (35). The vertical grid line corresponds to ${\lambda }_{1}^{2{N}_{1}}={\lambda }_{2}^{-2M}$, in this particular case, *λ*_1_ = 1/16. On the right side of this line the green and purple dashed lines corresponds to the two eigenvalues in Eq. (10). On the left side the green dashed line represents Eq. (9), the purple – Eq. (8). The horizontal grid line corresponds to $E={\lambda }_{2}^{-M}={4}^{-20}$.

The Ising-Kitaev chain split into two “topological” and one “trivial” region possesses two single-particle excitations (hereafter denoted by *k* = 1, 2) that are nearly degenerate with the ground state. As long as the parameters *λ*_*i*_ are not too close to unity and the sizes of the three regions are not too small, such that the three quantities ${\lambda }_{1}^{2{N}_{1}}$, ${\lambda }_{3}^{2{N}_{2}}$, and ${\lambda }_{2}^{-2M}$ are exponentially small, the energies and the wavefunctions of these states can be found analytically. Already the leading-order expression shows excellent agreement with the exact numerical diagonalization of the model as illustrated in Figs 3 and 4. The visible discrepancy between the analytic and numerical results for *λ*_1_ ~ 1 is to be expected: there the above parameters cease being exponentially small and the approximate analytic expressions become invalid.

Without specifying the relation between the three exponentially small parameters, even the leading-order expressions for the two eigenvalues ${ {\mathcal E} }_{1,2}$ are rather cumbersome. Therefore, here I focus on two representative limiting cases (the symbolic expression ${\mathscr{O}}({\lambda }^{N})$ hereafter denotes the omitted subleading, exponentially small terms).

#### Asymmetric chain

If the two “topological” regions of the chain are not symmetric, then compact expressions for the energies ${ {\mathcal E} }_{\mathrm{1,2}}$ can be found under following assumptions.

- “*Strong barrier*”. If ${\lambda }_{1}^{2{N}_{1}} > {\lambda }_{3}^{2{N}_{2}}\gg {\lambda }_{2}^{-2M}$, the two nearly zero-energy states are determined by the two “topological” regions of the chain, independently of the size of the “trivial” region. The first excited state has the energy
  6 $${ {\mathcal E} }_{1}=(1-{\lambda }_{3}^{2})\sqrt{\frac{{\lambda }_{2}^{2}-1}{{\lambda }_{2}^{2}-{\lambda }_{3}^{2}}}{\lambda }_{3}^{{N}_{2}}+{\mathscr{O}}({\lambda }^{N}),$$
  while the energy of the second excited state is
  7 $${ {\mathcal E} }_{2}=(1-{\lambda }_{1}^{2})\sqrt{\frac{{\lambda }_{2}^{2}-1}{{\lambda }_{2}^{2}-{\lambda }_{1}^{2}}}{\lambda }_{1}^{{N}_{1}}+{\mathscr{O}}({\lambda }^{N}),$$
  These results are illustrated in Fig. 3 by the dashed lines to the right of the vertical grid line (marking the end of the above parameter region ${\lambda }_{1}^{2{N}_{1}}={\lambda }_{2}^{-2M}$). Vanishing of the energies (6) and (7) at the point *λ*_1_ = *λ*_3_ = 1 is the artifact of the approximation. As the parameters *λ*_*i*_ approach unity, the approximate expressions reported here become invalid (while it is possible to write down exact expressions for ${ {\mathcal E} }_{\mathrm{1,2}}$ that are valid also near the critical point, their algebraic complexity renders them practically useless).
- “*Weak barrier*”. In the case ${\lambda }_{1}^{2{N}_{1}},{\lambda }_{3}^{2{N}_{2}}\ll {\lambda }_{2}^{-2M}$, the larger eigenvalue ${ {\mathcal E} }_{2}$ is determined by the “trivial” region of the chain

8 $${ {\mathcal E} }_{2}=({\lambda }_{2}^{2}-1)\sqrt{\frac{\mathrm{(1}-{\lambda }_{1}^{2})(1-{\lambda }_{3}^{2})}{({\lambda }_{2}^{2}-{\lambda }_{1}^{2})({\lambda }_{2}^{2}-{\lambda }_{3}^{2})}}\,{\lambda }_{2}^{-M}+{\mathscr{O}}({\lambda }^{N}),$$

while the energy of the lowest excited state is determined by the two “topological” regions combined

9 $${ {\mathcal E} }_{1}=\sqrt{(1-{\lambda }_{1}^{2})(1-{\lambda }_{3}^{2})}\,{\lambda }_{1}^{{N}_{1}}{\lambda }_{3}^{{N}_{2}}{\lambda }_{2}^{M}+{\mathscr{O}}({\lambda }^{N}).$$

These results are illustrated in Fig. 3 by the dashed lines on the left side of the vertical grid line.

#### Symmetric chain

In the symmetric case, ${\lambda }_{1}^{{N}_{1}}={\lambda }_{3}^{{N}_{2}}$, the two energies (7) and (6) coincide. In this case, one has to consider the subleading terms neglected so far, as the eigenvalues of the finite-size chain (2) are never truly degenerate.

- “*Strong barrier*”. Assuming ${\lambda }_{1}^{2{N}_{1}}\gg {\lambda }_{2}^{-2M}$, the resulting energies are given by
  10 $${{\mathscr{E}}}_{1(2)}^{{\rm{s}}{\rm{y}}{\rm{m}}}=(1-{\lambda }_{1}^{2})\sqrt{\frac{{\lambda }_{2}^{2}-1}{{\lambda }_{2}^{2}-{\lambda }_{1}^{2}}}{\lambda }_{1}^{{N}_{1}}[1\mp \frac{1}{2}{\lambda }_{1}^{-{N}_{1}}{\lambda }_{2}^{-M}\sqrt{\frac{{\lambda }_{2}^{2}-1}{{\lambda }_{2}^{2}-{\lambda }_{1}^{2}}}]+{\mathscr{O}}({\lambda }^{N}).$$
  This result is illustrated in Fig. 4 to the right of the vertical grid line (the exponentially small difference between the two energies (10) is indistinguishable on the scale of the plot).
- “*Weak barrier*”. In the limit, ${\lambda }_{1}^{2{N}_{1}}\ll {\lambda }_{2}^{-2M}$, no spurious degeneracy occurs and hence the expressions (8) and (9) are still valid, see Fig. 4 (to the left of the vertical grid line).

### Majorana zero modes

Elementary excitations of the model can be interpreted in terms of Majorana fermions^21, 40^. In fact, the fermionic form (2) of the Hamiltonian is already written in terms of the lattice Majorana fermions^40^

11 $${\hat{\zeta }}_{n}={\hat{c}}_{n}^{\dagger }+{\hat{c}}_{n},{\hat{\xi }}_{n}=-i({\hat{c}}_{n}^{\dagger }-{\hat{c}}_{n}).$$

In terms of the operators (11), the creation operator, ${\hat{\eta }}_{k}^{\dagger }$, of a single-particle excitation has the form [cf. Eq. (33)]

12 $${\hat{\eta }}_{k}^{\dagger }=\frac{1}{2}\sum _{n=1}^{L}[{\alpha }_{kn}{\hat{\zeta }}_{n}+i{\beta }_{kn}{\hat{\xi }}_{n}].$$

The two linear combinations

13 $${\hat{\gamma }}_{k}^{(\alpha )}=\sum _{n=1}^{L}{\alpha }_{kn}{\hat{\zeta }}_{n},\,{\hat{\gamma }}_{k}^{(\beta )}=\sum _{n=1}^{L}{\beta }_{kn}{\hat{\xi }}_{n},$$

are themselves Majorana operators^21^ in the sense that they satisfy the Majorana commutation relations

14 $$\{{\hat{\gamma }}_{k}^{(\alpha )},{\hat{\gamma }}_{k}^{(\beta )}\}=0,{({\hat{\gamma }}_{k}^{(\alpha )})}^{2}={({\hat{\gamma }}_{k}^{(\beta )})}^{2}=1.$$

The latter property follows from the fact that the vectors *α*_*kn*_ and *β*_*kn*_ are normalized and mutually orthogonal.

**Figure 5:**
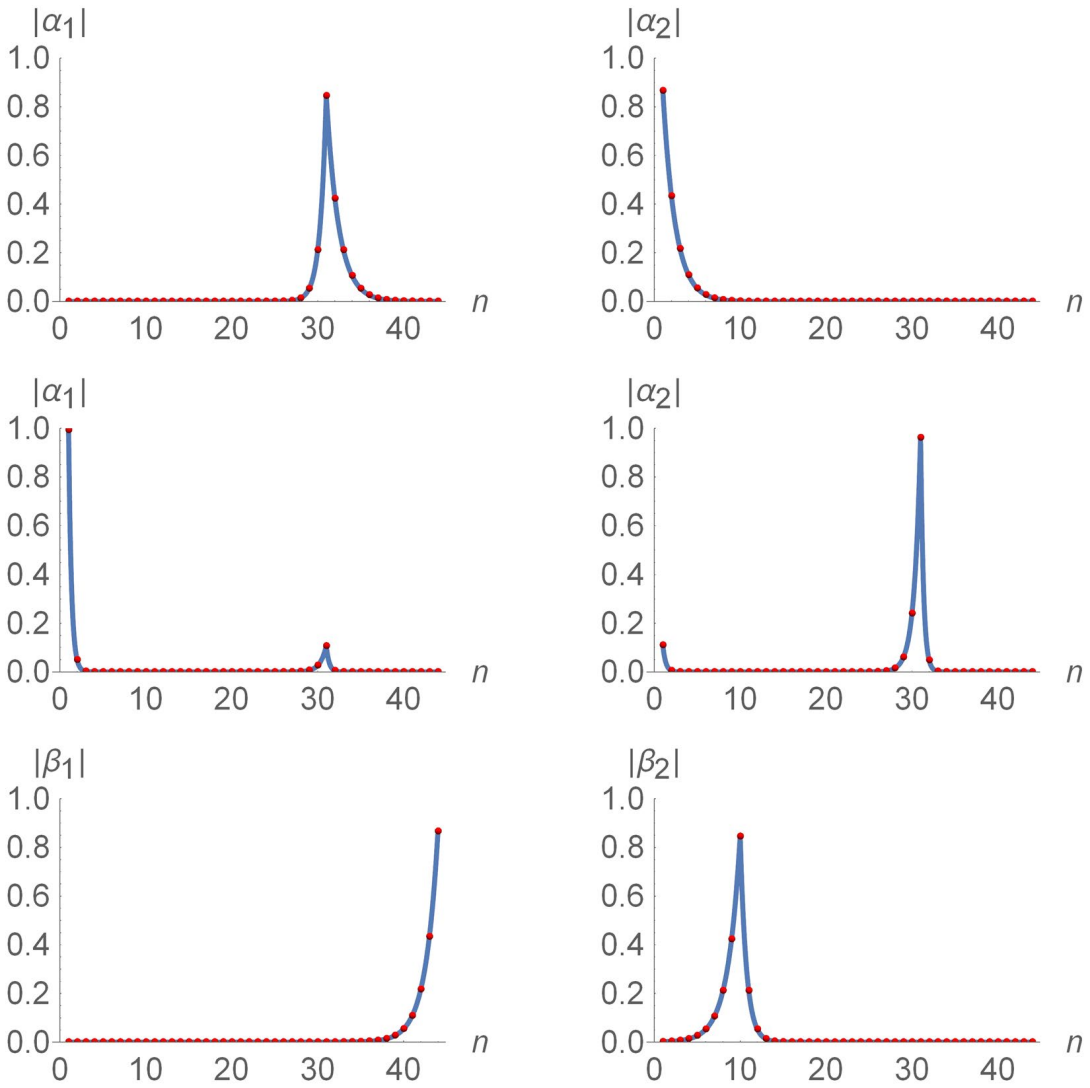
Majorana amplitudes of the two nearly zero-energy eigenstates of the Ising-Kitaev chain (1)–(2) with *N*_1_ = 10, *M* = 20, and *N*_2_ = 14 in the piece-wise uniform applied field (3). The red dots represent the result of the exact numerical diagonalization. The curves represent the analytic solutions. Top row: the amplitudes |*α*_1*n*_| and |*α*_1*n*_| in the strong barrier case, ${\lambda }_{1}={\lambda }_{3}=\mathrm{1/2}$, *λ*_2_ = 4. The curves are given in Eqs (15) and (17). Middle row: the amplitudes |*α*_1*n*_| and |*α*_1*n*_| in the weak barrier case, ${\lambda }_{1}={\lambda }_{3}=\mathrm{1/20}$, *λ*_2_ = 4, exhibiting weak delocalization. The curves are given in Eqs (18) and (19). Bottom row: the amplitudes |*β*_1*n*_| and |*β*_1*n*_| for ${\lambda }_{1}={\lambda }_{3}=\mathrm{1/2}$, *λ*_2_ = 4. The curves are given by either Eqs (15) and (17) or Eqs (18) and (19).

**Figure 6:**
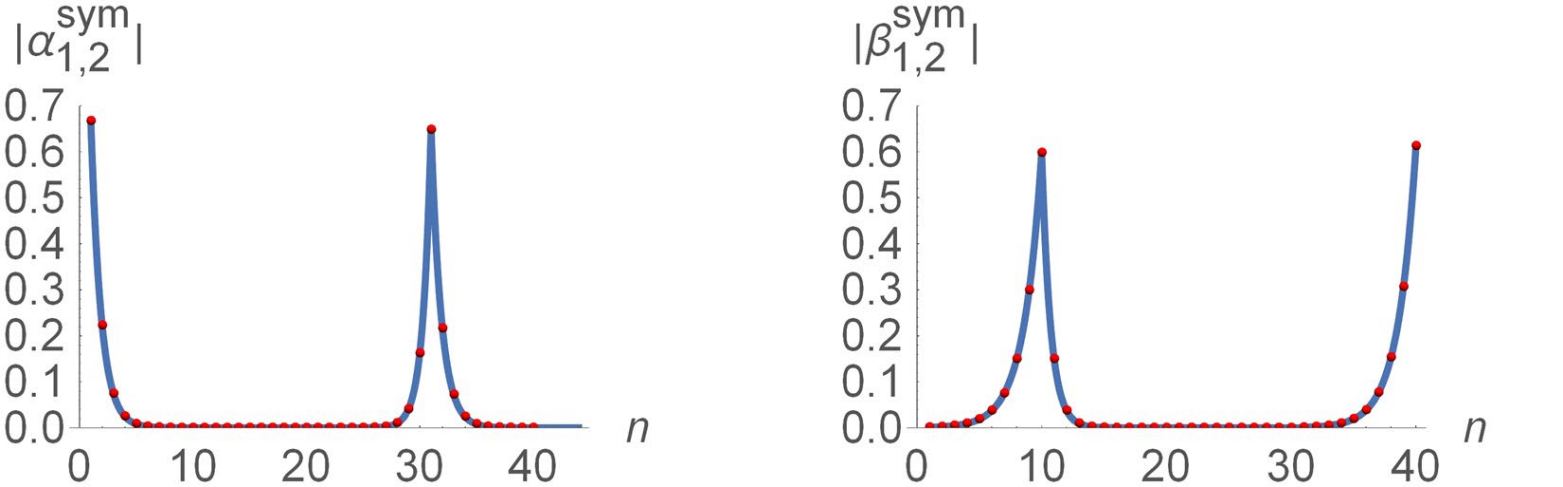
Majorana amplitudes (20) of the two nearly zero-energy eigenstates of the symmetric Ising-Kitaev chain (1)–(2) in the piece-wise uniform applied field (3) with ${\lambda }_{1}={\lambda }_{3}=\mathrm{1/2}$, *λ*_2_ = 4, *N*_1_ = *N*_2_ = 10, and *M* = 20. The red dots represent the result of the exact numerical diagonalization. The curves represent the analytic solutions given in Eq. (20).

By definition, the Majorana operators (13) are nonlocal linear combinations^21^ of the more conventional^40^ Majorana fermions (11). Typically, these combinations involve *all* sites of the chain^21^. However, for the two lowest excited states (7)–(10) the amplitudes *α*_1(2)*n*_ and *β*_1(2)*n*_ exhibit the exponential decay away from the interface points of the chain, allowing one to treat the nearly zero-energy Majorana states ${\hat{\gamma }}_{\mathrm{1(2)}}^{(\alpha )}$ and ${\hat{\gamma }}_{\mathrm{1(2)}}^{(\beta )}$ as essentially localized^21^, see Figs 5 and 6.

#### Asymmetric chain

- “*Strong barrier*”. In the limit ${\lambda }_{1}^{2{N}_{1}} > {\lambda }_{3}^{2{N}_{2}}\gg {\lambda }_{2}^{-2M}$, the leading behavior of the energy eigenvalues is given by Eqs (6) and (7). The corresponding amplitudes *α*_1(2)*n*_ and *β*_1(2)*n*_ can also be written in compact from, again retaining only the leading exponential terms. The first excited state (6) is characterized by the amplitudes
  15 $$\begin{array}{ccc}{\alpha }_{1n} & = & {(-1)}^{n}\{\begin{array}{cc}{\mathscr{O}}({\lambda }^{N}), & 1\leqslant n\leqslant {N}_{1},\\ {c}_{3}{\lambda }_{2}^{n-1-{N}_{1}-M}, & 1\leqslant n-{N}_{1}\leqslant M,\\ {c}_{3}{\lambda }_{3}^{n-1-{N}_{1}-M}, & 1+{N}_{1}+M\leqslant n\leqslant L,\end{array}\\ {\beta }_{1n} & = & {(-1)}^{n+1}\{\begin{array}{cc}{\mathscr{O}}({\lambda }^{N}), & 1\leqslant n\leqslant {N}_{1}+M,\\ {s}_{3}{\lambda }_{3}^{L-n}, & 1+{N}_{1}+M\leqslant n\leqslant L.\end{array}\end{array}$$
  where the symbolic expression ${\mathscr{O}}({\lambda }^{N})$ denoting the subleading terms is omitted in some lines for brevity and
  16 $${c}_{j}=\sqrt{\frac{\mathrm{(1}-{\lambda }_{j}^{2})({\lambda }_{2}^{2}-\mathrm{1)}}{{\lambda }_{2}^{2}-{\lambda }_{j}^{2}}},\,{s}_{j}=\sqrt{1-{\lambda }_{j}^{2}}.$$
  Hence with exponential accuracy, the lowest-energy excitation of the model can be described by the single fermion, ${\hat{\eta }}_{1}$, confined to the second “topological” region of the chain, cf. Eq. (12). The amplitudes (15) are illustrated in the two top panels in Fig. 5. The spread of the localized Majorana states over several lattice sites exhibited by Eq. (15) is a generic feature^21^ that can be seen also in the continuum limit^32^.
  The second eigenvalue (7) is characterized by the amplitudes
  17 $$\begin{array}{ccc}{\alpha }_{2n} & = & {(-1)}^{n-1}\{\begin{array}{cc}{s}_{1}{\lambda }_{1}^{n-1}+{\mathscr{O}}({\lambda }^{N}), & 1\leqslant n\leqslant {N}_{1},\\ {\mathscr{O}}({\lambda }^{N}), & 1\leqslant n-{N}_{1}\leqslant L,\end{array}\\ {\beta }_{2n} & = & {(-1)}^{n}\{\begin{array}{cc}{c}_{1}{\lambda }_{1}^{{N}_{1}-n}+{\mathscr{O}}({\lambda }^{N}), & 1\leqslant n\leqslant {N}_{1},\\ {c}_{1}{\lambda }_{2}^{{N}_{1}-n}+{\mathscr{O}}({\lambda }^{N}), & 1\leqslant n-{N}_{1}\leqslant M,\\ {\mathscr{O}}({\lambda }^{N}), & 1+{N}_{1}+M\leqslant n\leqslant L.\end{array}\end{array}$$
  The corresponding excitation ${\hat{\eta }}_{2}$ is confined to the first “topological” region of the chain. The amplitudes (17) are illustrated in the two bottom panels in Fig. 5.
  The results (15) and (17) confirm that in the limit ${\lambda }_{1}^{2{N}_{1}} > {\lambda }_{3}^{2{N}_{2}}\gg {\lambda }_{2}^{-2M}$ the two lowest-energy excitations of the model behave similarly to those of the two independent “topological” regions. In particular, they exhibit four nearly zero-energy Majorana fermions localized at the edges of the “topological” regions.
- “*Weak barrier*”. In the limit, ${\lambda }_{1}^{2{N}_{1}},{\lambda }_{3}^{2{N}_{2}}\ll {\lambda }_{2}^{-2M}$, the structure of the wave-functions of the two lowest excited states is significantly different. The first excited state (6) is characterized by the amplitudes

18 $$\begin{array}{ccc}{\alpha }_{1n} & = & {(-1)}^{n}{s}_{1}\{\begin{array}{cc}{\lambda }_{1}^{n-1}, & 1\leqslant n\leqslant {N}_{1},\\ {\lambda }_{1}^{{N}_{1}}{\lambda }_{2}^{n-1-{N}_{1}}, & 1\leqslant n-{N}_{1}\leqslant M,\\ {\lambda }_{1}^{{N}_{1}}{\lambda }_{2}^{M}{\lambda }_{3}^{n-1-{N}_{1}-M}, & 1+{N}_{1}+M\leqslant n\leqslant L,\end{array}\\ {\beta }_{1n} & = & {(-1)}^{n+1}\{\begin{array}{cc}{\mathscr{O}}({\lambda }^{N}), & 1\leqslant n\leqslant {N}_{1}+M,\\ {s}_{3}{\lambda }_{3}^{L-n}, & 1+{N}_{1}+M\leqslant n\leqslant L,\end{array}\end{array}$$

The amplitude *β*_1*n*_ is identical with Eq. (15), but the amplitude *α*_1*n*_ has changed. In the first “topological” region of the chain, it behaves as the corresponding amplitude of the second excited state (17) of the strong barrier case. Moreover, there is a nonzero probability to find this quasiparticle also at the interface between the “trivial” and the second “topological” regions, see Fig. 5, i.e. the corresponding Majorana fermion is essentially “delocalized” between the two points.

The “delocalization” of the Majorana amplitude *α*_1*n*_ in Eq. (18) is rather weak. For the following choice of values of the parameters (4), ${\lambda }_{1}={\lambda }_{3}=\mathrm{1/20}$, *λ*_2_ = 4, and the sizes of the chain segments *N*_1_ = 10, *N*_2_ = 14, *M* = 20, the peak values of the amplitude *α*_1*n*_ are $|{\alpha }_{\mathrm{1,1}}|=0.999$ and $|{\alpha }_{\mathrm{1,31}}|=0.107$. Whether this feature survives the thermodynamic limit depends on what happen to the value of the product of a small and large parameters ${\lambda }_{1}^{{N}_{1}}{\lambda }_{2}^{M}$ in the limiting procedure.

The second eigenvalue (7) is characterized by the amplitudes

19 $$\begin{array}{ccc}{\alpha }_{2n} & = & {(-1)}^{n}\{\begin{array}{cc}-({s}_{1}^{2}/{c}_{3}){\lambda }_{2}^{M}{\lambda }_{1}^{{N}_{1}+n-1}, & 1\leqslant n\leqslant {N}_{1},\\ {c}_{3}{\lambda }_{2}^{n-1-{N}_{1}-M}, & 1\leqslant n-{N}_{1}\leqslant M,\\ {c}_{3}{\lambda }_{3}^{n-1-{N}_{1}-M}, & 1+{N}_{1}+M\leqslant n\leqslant L,\end{array}\\ {\beta }_{2n} & = & {(-1)}^{n}\{\begin{array}{cc}{c}_{1}{\lambda }_{1}^{{N}_{1}-n}, & 1\leqslant n\leqslant {N}_{1},\\ {c}_{1}{\lambda }_{2}^{{N}_{1}-n}, & 1\leqslant n-{N}_{1}\leqslant M,\\ {\mathscr{O}}({\lambda }^{N}), & 1+{N}_{1}+M\leqslant n\leqslant L.\end{array}\end{array}$$

Again, the amplitude *β*_2*n*_ remains the same as in Eq. (17), while the amplitude *α*_1*n*_ exhibits the above “delocalization”. For the same choice of parameters (${\lambda }_{1}={\lambda }_{3}=\mathrm{1/20}$, *λ*_2_ = 4 and *N*_1_ = 10, *N*_2_ = 14, *M* = 20), I find $|{\alpha }_{\mathrm{2,1}}|=0.967$ and $|{\alpha }_{\mathrm{2,31}}|=0.111$.

In contrast to the strong barrier case, the wave-function of lowest-energy fermion ${\hat{\eta }}_{1}$ is now mostly spread between the two outer edges of the chain, with a small weight at the interface between the “trivial” and the second “topological” region. The second excitation ${\hat{\eta }}_{2}$ is mostly confined to the edges of the “trivial” region, with the small weight at the beginning of the chain, see Fig. 5.

#### Symmetric chain

- The two lowest-energy excitations of the symmetric chain with *the strong barrier*, ${\lambda }_{1}^{2{N}_{1}}={\lambda }_{3}^{2{N}_{2}}\gg {\lambda }_{2}^{-2M}$, are characterized by the amplitudes, see Fig. 6 (the symbolic expression ${\mathscr{O}}({\lambda }^{N})$ denoting the subleading terms is omitted for brevity)
  20 $$\begin{array}{ccc}{\alpha }_{1(2),n}^{{\rm{s}}{\rm{y}}{\rm{m}}} & = & \frac{{(-1)}^{n}}{\sqrt{2}}\{\begin{array}{cc}\mp \sqrt{1-{\lambda }_{1}^{2}}{\lambda }_{1}^{n-1}, & 1\leqslant n\leqslant {N}_{1},\\ {c}_{1}{\lambda }_{2}^{n-1-{N}_{1}-M}, & 1\leqslant n-{N}_{1}\leqslant M,\\ {c}_{1}{\lambda }_{1}^{n-1-{N}_{1}-M}, & 1+{N}_{1}+M\leqslant n\leqslant L,\end{array}\\ {\beta }_{1(2),n}^{{\rm{s}}{\rm{y}}{\rm{m}}} & = & {(-1)}^{n}\{\begin{array}{cc}\mp {c}_{1}{\lambda }_{1}^{{N}_{1}-n}, & 1\leqslant n\leqslant {N}_{1},\\ \mp {c}_{1}{\lambda }_{2}^{{N}_{1}-n}, & 1\leqslant n-{N}_{1}\leqslant M,\\ \sqrt{1-{\lambda }_{1}^{2}}{\lambda }_{1}^{L-n}, & 1+{N}_{1}+M\leqslant n\leqslant L.\end{array}\end{array}$$
  In this case, the excitations ${\hat{\eta }}_{\mathrm{1,2}}^{\dagger }$ are no longer confined to one of the two “topological” regions of the chain, but are spread symmetrically over both of them.
- In the *weak barrier* case, the amplitudes *α*_*n*_ and *β*_*n*_ are still described by Eqs (18) and (19).

The exponential decay of the amplitudes (15)–(20) can be described in terms of a correlation length, which is specific to each of the three regions of the chain

21 $${\ell }_{0i}\sim \mathrm{1/}|\mathrm{ln}\,{\lambda }_{i}|.$$

In experiments on discreet systems^33–36^, the realistic values of *λ*_*i*_ might not be extreme and hence the correlation lengthes (21) might not be very small. In such case, even the localized Majorana fermions are spread over several lattice sites as illustrated in Figs 5 and 6.

### Effective low energy theory

Applications to quantum computation^27^ involve adiabatic manipulations of the Majorana zero modes. This means that any external perturbation applied to the system should be slow enough to avoid exciting higher-energy gapped states. The remaining low-energy sector of the theory consists of the ground state |*GS*〉 and the nearly degenerate excitations that can be interpreted in terms of Majorana zero modes.

For the specific model considered in this paper, the low-energy sector contains four states

22 $$|GS\rangle ,\,{\eta }_{1}^{\dagger }|GS\rangle ,\,{\eta }_{2}^{\dagger }|GS\rangle ,\,{\eta }_{1}^{\dagger }{\eta }_{2}^{\dagger }|GS\rangle ,$$

where the last state is the two-particle excitation. These four states can be further split into two groups of mutually orthogonal states, belonging to the two parity sectors of the model where the total fermion number is either even or odd.

Projecting the Hamiltonian (2) onto either of the above sectors, one finds the effective low-energy theory. In the one-fermion (odd) sector, the effective Hamiltonian has the simplest form in the basis of the Majorana fermions ${\hat{\gamma }}_{\mathrm{1(2)}}^{(\alpha )}$ and ${\hat{\gamma }}_{\mathrm{1(2)}}^{(\beta )}$.

- In the *asymmetric chain* with the *strong barrier*, i.e. in the limit ${\lambda }_{1}^{2{N}_{1}} > {\lambda }_{3}^{2{N}_{2}}\gg {\lambda }_{2}^{-2M}$, the localized Majorana fermions describe the exact eigenstates of the model. Hence, the projected Hamiltonian in the basis the four Majorana states (counted from left to right) has the block-diagonal structure
  23 $${\hat{H}}_{{\rm{e}}{\rm{f}}{\rm{f}}}\propto (\begin{array}{cccc}0 & -i{{\mathscr{E}}}_{2} & 0 & 0\\ i{{\mathscr{E}}}_{2} & 0 & 0 & 0\\ 0 & 0 & 0 & -i{{\mathscr{E}}}_{1}\\ 0 & 0 & i{{\mathscr{E}}}_{1} & 0\end{array}).$$
  Note the absence of any coupling between the two pairs of the Majorana zero modes, *γ*_2_, *γ*_3_ and *γ*_1_, *γ*_4_ (since the exact orthogonal eigenstates of the model are described by *γ*_1_, *γ*_2_ and *γ*_3_, *γ*_4_).
- In contrast, in the case of the *symmetric chain* with the *strong barrier*, ${\lambda }_{1}^{2{N}_{1}}={\lambda }_{3}^{2{N}_{2}}\gg {\lambda }_{2}^{-2M}$, the Majorana amplitudes (20) are not localized at single interface points in the chain. One can still represent the effective Hamiltonian in the basis of the four localized Majorana fermions. However, now these objects are no longer associated with the exact eigenstates and hence additional couplings appear. Introducing a short-hand notation for the eigenvalues (10)
  24 $${ {\mathcal E} }_{\mathrm{1(2)}}^{{\rm{sym}}}=\varepsilon \pm \delta ,\,\delta \ll \varepsilon ,$$
  I find the following Hamiltonian (using an obvious basis rotation)
  25 $${\hat{H}}_{{\rm{e}}{\rm{f}}{\rm{f}}}\propto (\begin{array}{cccc}0 & -i\varepsilon  & 0 & -i\delta \\ i\varepsilon  & 0 & -i\delta  & 0\\ 0 & i\delta  & 0 & -i\varepsilon \\ i\delta  & 0 & i\varepsilon  & 0\end{array}).$$
  The Hamiltonian (25) is no longer block-diagonal: the two pairs of the Majorana zero modes, *γ*_2_, *γ*_3_ and *γ*_1_, *γ*_4_, are now coupled. The effective coupling of both pairs is the same (given by *δ*) despite the large difference in their respective separation. This result is the consequence of the nonlocal nature of the eigenstates of the model.
- In the *weak barrier* case, the low-energy excitations are no longer confined to the “topological” regions of the chain. In order to take into account the “delocalization” of the amplitudes *α*_1(2)*n*_, see Eqs (18) and (19), I denote the ratio of the peak values of *α*_1(2)*n*_ as $\kappa \approx {\lambda }_{1}^{{N}_{1}}{\lambda }_{2}^{M}s\mathrm{1/}c3\ll 1$ (up to exponentially small corrections) and find the low-energy Hamiltonian (in the same basis of *γ*_*i*_ counted from left to right)

26 $${\hat{H}}_{{\rm{e}}{\rm{f}}{\rm{f}}}\propto (\begin{array}{cccc}0 & -i\kappa {{\mathscr{E}}}_{2} & 0 & -i{{\mathscr{E}}}_{1}\\ i\kappa {{\mathscr{E}}}_{2} & 0 & -i{{\mathscr{E}}}_{2} & 0\\ 0 & i{{\mathscr{E}}}_{2} & 0 & -i\kappa {{\mathscr{E}}}_{1}\\ i{{\mathscr{E}}}_{1} & 0 & i\kappa {{\mathscr{E}}}_{1} & 0\end{array}).$$

In contrast to Eqs (23) and (25), the leading terms in Eq. (26) are confined to the antidiagonal. This results reflects the structure of the single-fermion excitations (18) and (19): the lowest-energy excitation (18) is mostly spread between the two outer edges of the chain (and is described by *γ*_1_, *γ*_4_), while the fermion (19) – to the edges of the “trivial” region (corresponding to *γ*_2_, *γ*_3_). Neglecting weak “delocalization” completely (i.e. in the limit *κ* → 0), the Hamiltonian (26) can be made block-diagonal by renumbering the localized Majorana operators.

### Kitaev chain with a T-junction

**Figure 7:**
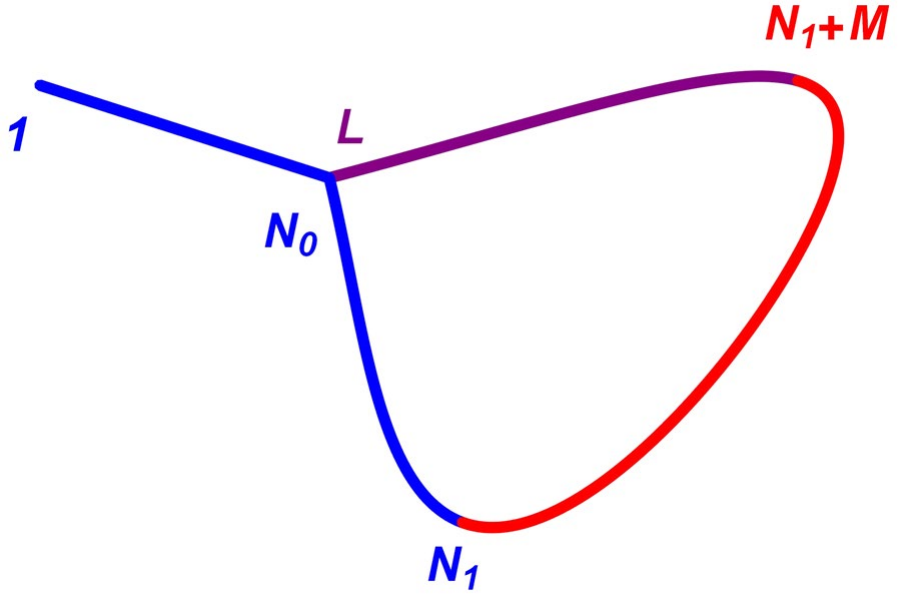
The nonuniform Ising-Kitaev chain with a T-junction. The chain contains two “topological” (blue and purple) regions and one “trivial” (red) region. The first “topological” region is characterized by the parameter *λ*_1_ < 1 and contains the first *N*_1_ sites. The next *M* sites are occupied by the “trivial” phase with *λ*_2_ > 1. The remaining portion of the chain of the length ${N}_{2}=L-{N}_{1}-M$ is occupied by the second “topological” region with *λ*_3_ < 1. The T-junction is located at the site *N*_0_. In this paper, I report results for the case where the T-junction is approximately in the middle of the first “topological” region of the chain.

Consider now a modified model where one of the chain ends is coupled to an intermediate site forming a T-junction (sometimes also referred to as a Y-junction), see Fig. 7. Such a model can be easily formulated in the fermion language by adding another coupling term to the Hamiltonian (2)

27 $$\hat{H}=-J\sum _{n=1}^{L-1}({\hat{c}}_{n}^{\dagger }-{\hat{c}}_{n})({\hat{c}}_{n+1}^{\dagger }+{\hat{c}}_{n+1})-J({\hat{c}}_{L}^{\dagger }-{\hat{c}}_{L})({\hat{c}}_{{N}_{0}}^{\dagger }+{\hat{c}}_{{N}_{0}})-\sum _{n=1}^{L}{h}_{n}({\hat{c}}_{n}^{\dagger }-{\hat{c}}_{n})({\hat{c}}_{n}^{\dagger }+{\hat{c}}_{n}),1 < {N}_{0} < L.$$

A similar modification is also possible for the Ising Hamiltonian (1). However, such model cannot be mapped onto a quadratic fermionic Hamiltonian similar to (27) due to non-cancellation of the Jordan-Wigner strings at the junction point.

T-junctions play an important role in the literature on Majorana-based quantum computation^27, 49–51^, where they are the key elements of connected networks of quantum wires that are envisioned to allow for braiding operations. A discussion of braiding as well as any other time-dependent processes involving Majorana fermions is beyond the scope of this paper.

The Hamiltonian (27) is quadratic and can be diagonalized by the same exact method as the model (2). Due to the more complicated topology and larger number of parameters, the model (27) exhibits many more parameter regimes than the chain (2). A comprehensive discussion of all of these regimes will be presented elsewhere. In this paper, I focus on a single parameter regime, where the nearly zero-energy eigenstates are described by the amplitudes *α*_*n*_ that are delocalized between two interface points similarly to Eq. (20). In particular, I consider the regime, where the junction site *N*_0_ is approximately in the middle of its “topological” region, which is twice as long as the second “topological” region. Choosing the equal parameters *λ*_1_ = *λ*_3_ < 1 describing the “topological” regions, I achieve a configuration that is somewhat analogous to the symmetric chain.

**Figure 8:**
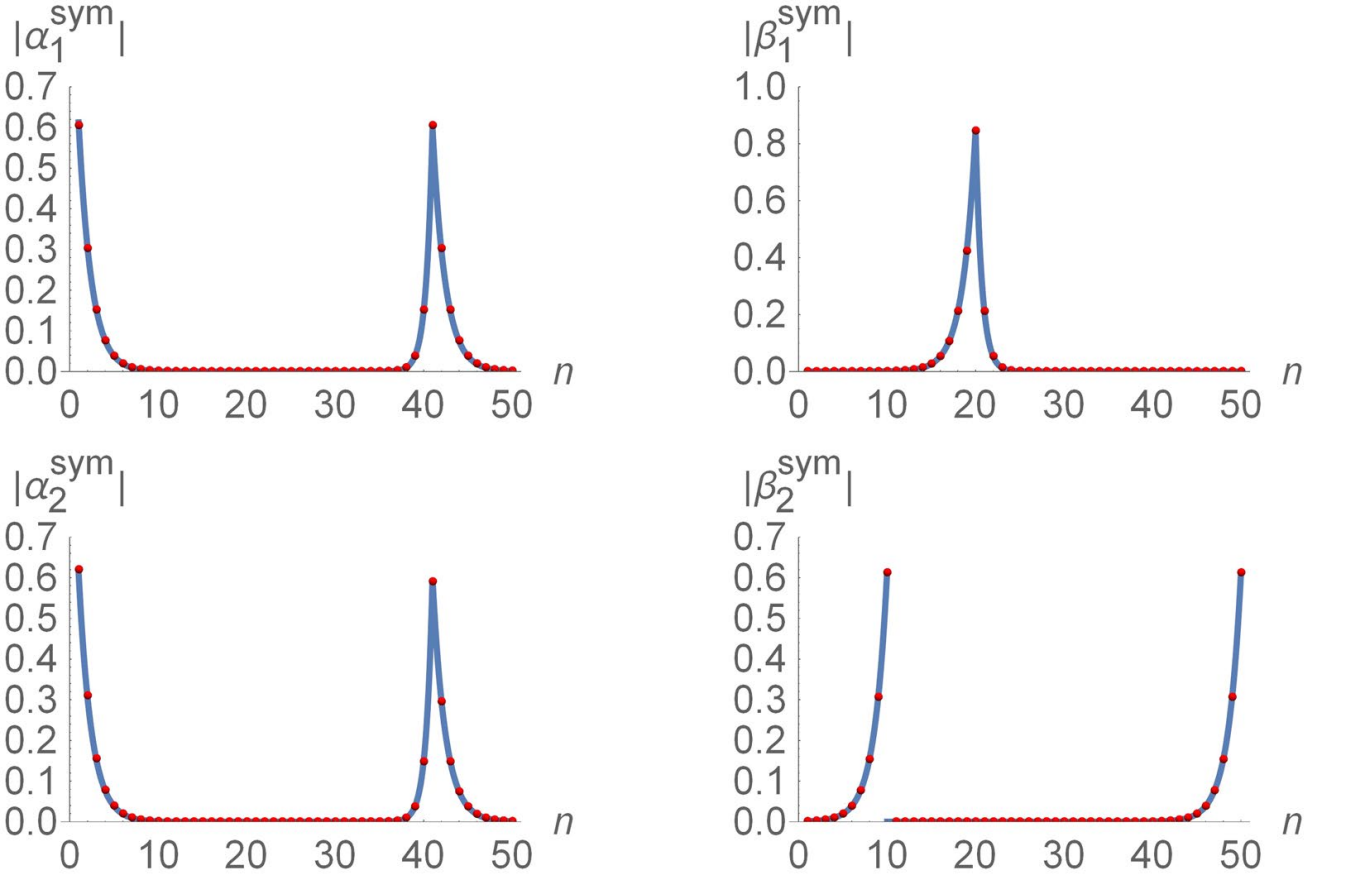
Majorana amplitudes of the two nearly zero-energy eigenstates of the modified Kitaev chain (27) with a T-junction in the piece-wise uniform applied field (3) with ${\lambda }_{1}={\lambda }_{3}=\mathrm{1/2}$, *λ*_2_ = 4, *N*_1_ = *M* = 20, *N*_2_ = 10, and *N*_0_ = 11. The red dots represent the result of the exact numerical diagonalization. The solid curves are presented for comparison. The amplitudes ${\alpha }_{\mathrm{1,2}}^{{\rm{sym}}}$ shown on the left two panels are compared with Eq. (20). The amplitude ${\beta }_{1}^{{\rm{sym}}}$ appears to be well described by Eq. (17). The amplitude ${\beta }_{2}^{{\rm{sym}}}$ of the Majorana zero mode localized at the junction point is given by Eq. (28).

The results of the exact numerical diagonalization of the model (27) in the chosen parameter regime (with *N*_1_ = 20, *M* = 20, *N*_2_ = 10, and *N*_0_ = 11) are presented in Fig. 8 by the red dots and illustrated in Fig. 1. The solution is characterized by the amplitudes ${\alpha }_{\mathrm{1,2}}^{{\rm{sym}}}$ that are delocalized between the edge point of the chain and one of the borders of the “trivial” region. The amplitude ${\alpha }_{2}^{{\rm{sym}}}$ appears to be in perfect agreement with Eq. (20), while the amplitude ${\alpha }_{1}^{{\rm{sym}}}$ shows a barely perceptible deviation (see the solid curves in the two left panels in Fig. 8).

Now the amplitudes ${\beta }_{\mathrm{1,2}}^{{\rm{sym}}}$ are no longer delocalized. The amplitude ${\beta }_{1}^{{\rm{sym}}}$ is localized at the edge of the “trivial” region and is perfectly described by Eq. (17), see the top right panel in Fig. 8. The amplitude ${\beta }_{2}^{{\rm{sym}}}$ is localized at the T-junction point, see the bottom right panel in Fig. 8, and can be described analytically by

28 $${\beta }_{2n}^{{\rm{s}}{\rm{y}}{\rm{m}}}=\frac{{(-1)}^{n}}{\sqrt{2}}\{\begin{array}{cc}{s}_{1}{\lambda }_{1}^{{N}_{0}-1-n}+{\mathscr{O}}({\lambda }^{N}), & 1\leqslant n\leqslant {N}_{0}-1,\\ {\mathscr{O}}({\lambda }^{N}), & {N}_{0}\leqslant n\leqslant {N}_{1}+M,\\ -{s}_{1}{\lambda }_{1}^{L-n}+{\mathscr{O}}({\lambda }^{N}), & {N}_{1}+M+1\leqslant n\leqslant L.\end{array}$$

The asymmetry of this amplitude – ${\beta }_{2}^{{\rm{sym}}}$ is spread over only two out of the three branches of the junction – is related to the chirality of the junction^50^ described by the explicitly asymmetric Hamiltonian (27), as well as to the time reversal symmetry^52^ of the Hamiltonian (27).

## Discussion

In this paper I presented the exact analytic solution of the nonuniform Ising-Kitaev chain with open boundary conditions. The motivation for this work was two-fold: (i) I was motivated by the proposal^33, 34^ of experimental realization of zero-energy Majorana states in an artificial spin chain engineered using Josephson qubits; such a system would be discreet and, given current technological limitations, contain not too many qubits; (ii) I wanted to reach a better understanding of the the effective coupling between the Majorana zero modes in networked systems used as paradigmatic examples of possible applications to quantum computing^27^, in particular, the common “nearest-neighbor” form of the effective low-energy Hamiltonian^30, 47, 48^. The model solved in this paper represents the first step in reaching these goals. In the case, where the two “topological” regions of the chain are separated by a “trivial” region, the exact analytic form of the eigenvalues and eigenvectors can be found. Moreover, the resulting low-energy theory does not support the “nearest-neighbor” approach^30, 47, 48^.

Indeed, in the generic parameter regime with the “strong barrier”, the single-fermion sector of the effective low-energy theory contains two single-fermion states, each confined to its own “topological” region as if these regions were disconnected. Similarly to the original Kitaev model^21^, each of these states can be interpreted in terms of two Majorana states localized at the edges of the “topological” regions, as expected^27, 30, 47, 48^. In the basis of the localized Majorana fermions, the effective Hamiltonian has the block-diagonal form (23). Here the two pairs of Majorana fermions *γ*_1_, *γ*_2_ and *γ*_3_, *γ*_4_ form the two single-fermion eigenstates. The orthogonality of the single-fermion eigenstates leads to the absence of any coupling between *γ*_2_ and *γ*_3_, that is typically included in the “nearest-neighbor” approach^30, 47, 48^.

In contrast, in the specially fine-tuned case of the symmetric chain the single-fermion eigenstates are equally spread between the two “topological” regions. Consequently, the corresponding Majorana fermions are localized not at one, but at two separate interface points. While the resulting low-energy Hamiltonian can of course be represented in the above basis of localized Majorana states, the latter are no longer related to the exact eigenstates of the model. As a result, the Hamiltonian (24) exhibits additional couplings between the two pairs *γ*_2_, *γ*_3_ and *γ*_1_, *γ*_4_. These couplings are identical despite the large difference in the separation between *γ*_2_, *γ*_3_ and *γ*_1_, *γ*_4_. Again, this contradicts the “nearest-neighbor” approach^30, 47, 48^, where the coupling between *γ*_1_, *γ*_4_ is not included due to the larger separation (as compared to other pairs of Majorana fermions).

Now, in the case of the weak barrier the amplitudes (18) and (19) also exhibit “delocalization” between two interface points (although to a significantly lesser degree). Here, the dominant terms in the effective Hamiltonian (26) are the couplings between *γ*_2_, *γ*_3_ and *γ*_1_, *γ*_4_, while the couplings *γ*_1_, *γ*_2_ and *γ*_3_, *γ*_4_ appear with the typically small coefficient *κ* (due to weak “delocalization”). As a result, the Hamiltonian (26) is also incompatible with the “nearest-neighbor” form^30, 47, 48^.

Finally, “delocalization” of the Majorana zero modes between two separated interface points is not an artifact^53^ of the model (2). In particular, the modified model (27) also exhibits this “delocalization” (again, requiring some fine-tuning).

The results of this paper are relevant for experimentalists designing small systems hosting multiple zero-energy Majorana states^33–36^. In particular, in systems involving relatively few Josephson qubits with conservative parameter values the spreading of the Majorana zero modes over a few qubits and their “delocalization” between two well separated points are generic effects^53^ that need to be taken into account while interpreting the experimental data and especially when planning any kind of manipulation of the Majorana bound states by some external bias. “Delocalization” of the Majorana zero modes and the corresponding separation-independent effective coupling between some pairs of the localized Majorana fermions could be observed in experimental nanowire samples in the presence of additional symmetries incorporated into the system design.

## Methods

An arbitrary quadratic Hamiltonian, including Eqs (2) and (27), can be written in the following form (where *A*_*ij*_ and *B*_*ij*_ are symmetric and antisymmetric *L* × *L* matrices, respectively)

29 $$\hat{H}=-2J\sum _{i,j}[{\hat{c}}_{i}^{\dagger }{A}_{ij}{\hat{c}}_{i}+\frac{1}{2}({\hat{c}}_{i}^{\dagger }{B}_{ij}{\hat{c}}_{j}^{\dagger }+h.c.)].$$

The Hamiltonian (29) can be diagonalized exactly using the method^39^ suggested by Lieb, Schultz, and Mattis for the 1D XY model^42^ and used by Pfeuty to solve the uniform quantum Ising model^43^ (*h*_*n*_ = *h*). The method is well known in the theory of superconductivity^46^ and is based on the Bogolyubov transformation

30 $${\hat{\eta }}_{k}=\sum _{n}({g}_{kn}{\hat{c}}_{n}+{h}_{kn}{\hat{c}}_{n}^{\dagger }),\,{\hat{\eta }}_{k}^{\dagger }=\sum _{n}({g}_{kn}{\hat{c}}_{n}^{\dagger }+{h}_{kn}{\hat{c}}_{n}),$$

where *g*_*kn*_ and *h*_*kn*_ are real coefficients and the resulting operators ${\hat{\eta }}_{k}$ satisfy fermionic commutation relations. The latter requirement leads to the fact that the coefficients *g*_*kn*_ and *h*_*kn*_ form a complete, orthonormal basis in the *L*-dimensional Euclidean vector space.

The coefficients *g*_*kn*_ and *h*_*kn*_ of the Bogolyubov transformation (30) can be found by assuming the diagonal form of the Hamiltonian in terms of the operators ${\hat{\eta }}_{k}$ and using the commutation relations

$$[{\hat{\eta }}_{k},\hat{H}]={{\mathscr{E}}}_{k}{\hat{\eta }}_{k}.$$

Using the explicit expressions (29) and (30), one finds for the following relations for the linear combinations of *g*_*kn*_ and *h*_*kn*_

31 $$\begin{array}{rcl}{ {\mathcal E} }_{k}{\alpha }_{kj} & = & -\sum _{i}{\beta }_{ki}({A}_{ij}+{B}_{ij}),\\ { {\mathcal E} }_{k}{\beta }_{kj} & = & -\sum _{i}{\alpha }_{ki}({A}_{ij}-{B}_{ij}),\\ { {\mathcal E} }_{k}^{2}{\alpha }_{kj} & = & \sum _{i}{\alpha }_{ki}(A-B)(A+B{)}_{ij},\end{array}$$

where

32 $${\alpha }_{kn}={g}_{kn}+{h}_{kn},\,{\beta }_{kn}={g}_{kn}-{h}_{kn}.$$

The coefficients *α*_*kj*_ form the eigenvectors of the real, symmetric matrix (*A* − *B*) (*A* + *B*) and hence can be chosen to form a real, orthonormal set. Then for nonzero eigenvalues, ${ {\mathcal E} }_{k}\ne 0$, the coefficients *β*_*kj*_ are normalized automatically. For ${ {\mathcal E} }_{k}=0$, one can normalize *β*_*kj*_. The resulting creation operators of the Bogolyubov fermions are given by

33 $${\hat{\eta }}_{k}^{\dagger }=\frac{1}{2}\sum _{n=1}^{L}[{\alpha }_{kn}({\hat{c}}_{n}^{\dagger }+{\hat{c}}_{n})+{\beta }_{kn}({\hat{c}}_{n}^{\dagger }-{\hat{c}}_{n})].$$

In the particular case of the Hamiltonian (2), the resulting diagonal form is given by Eq. (5).

The outlined diagonalization procedure is applicable to any quadratic Hamiltonian in any dimensionality and allows for an efficient numerical calculation of the eigenvalues ${ {\mathcal E} }_{k}$ and the coefficients *α*_*kj*_ and *β*_*kj*_ with arbitrary precision. However, analytic solution is manageable only in a few relatively simple cases. Fortunately, the model (2) with the specific choice (3) of the applied fields is one of them. The exact single-particle energies ${ {\mathcal E} }_{k}$ of this model can be expressed as

34 $${ {\mathcal E} }^{2}=1+{\lambda }_{1}^{2}+2{\lambda }_{1}\,\cos \,{\vartheta }_{1}=1+{\lambda }_{2}^{2}+2{\lambda }_{2}\,\cos \,{\vartheta }_{2}=1+{\lambda }_{3}^{2}+2{\lambda }_{3}\,\cos \,{\vartheta }_{3},$$

in terms of nontrivial solutions, ${\vartheta }_{i}$, to the equation

35 $${\lambda }_{1}{D}_{1}({N}_{1}){D}_{2}(M,{N}_{2})={\lambda }_{2}{D}_{1}({N}_{1}+1){D}_{2}(M+1,{N}_{2}),$$

where

36 $$\begin{array}{rcl}\,\,\,{D}_{1}({N}_{1}) & = & {\lambda }_{1}\,\sin \,{N}_{1}\,{\vartheta }_{1}+\,\sin ({N}_{1}-1){\vartheta }_{1},\\ {D}_{2}(M,{N}_{2}) & = & {\lambda }_{3}\,\sin ({N}_{2}+1){\vartheta }_{3}\,\sin \,M{\vartheta }_{2}-{\lambda }_{2}\,\sin \,{N}_{2}\,{\vartheta }_{3}\,\sin (M-1){\vartheta }_{2}.\end{array}$$

The latter equalities in Eq. (34) provide additional constraints on ${\vartheta }_{i}$, which guarantee the uniqueness of the solution.

Consider now the energy (34) as a function of ${\vartheta }_{i}$, regardless of which values of ${\vartheta }_{i}$ are allowed by Eq. (35). For real ${\vartheta }_{i}$, this function exhibits a minimum at ${\vartheta }_{i}=\pi$. The minimum value of the energy gives a reasonable lower bound for the bulk gap

37 $${\rm{\Delta }}\approx 2J\mathop{{\rm{\min }}}\limits_{i=1,2,3}|1-{\lambda }_{i}|.$$

Hence, any subgap states including nearly zero-energy states are described by complex solutions to Eq. (35).
